# Medical Opinion and Movement

**Published:** 1910-02-12

**Authors:** 


### Medical Opinion and Movement.

### Congenital Infantile Stridor.

Opinions are still much divided as to the cause of Congenital Infantile Stridor. Some agree with Hochsinger in believing the condition to be due to hypertrophy of the thymus gland; others, and among them Professor Jokay, think that deformity of the epiglottis has much to do with the stridor. This latter has recently published in the *Archives de Médicine des Enfants* three cases of the condition in which enlargement of the thymus gland could have had no causal influence. In the first case, that of a female infant of sixteen months with marked symptoms of inspiratory stridor, palpation of the epiglottis showed this structure to be rolled up in its long axis. Intubation afforded marked relief, and no enlargement of the thymus could be made out either by percussion or by radiography. In the second case, that of an infant aged six weeks, a similar condition of the epiglottis was discovered. This child died, and at the autopsy the presence of the deformity was confirmed, there being, moreover, no enlargement of the thymus. The third observation concerned an infant of three months. Palpation of the epiglottis revealed a similar deformity. Laryngoscopy under chloroform showed that with a moderate degree of stridor the left half of the epiglottis applied itself to the opening of the larynx at each inspiration. During a marked spasm the whole of the epiglottis was aspirated towards the interior of the larynx, such aspiration being accompanied by the characteristic sound of laryngo spasm. In this case again radioscopy revealed no enlargement of the thymus.

### Puerperal Septicæmia.

An interesting article by Labusquière in *Les Annales de Gynécologie et d'Obstétrique* summarises some recent work with reference to the Streptococcus and Hæmolysis in Obstetrics. The diagnosis and prognosis of puerperal infection could be established from a scientific standpoint, if only some distinctive characteristic were known whereby a virulent streptococcus could be recognised from the numerous inoffensive varieties which inhabit the female genital tract. Schottlander believes that such a character exists, and that it consists in the hæmolytic power of the pathogenic streptococcus. When grown on blood-agar this variety causes hæmolysis, the degree of virulence of the organism being always proportional to the intensity of the blood destruction. Saprophytic types of this organism have not this hæmolytic power. This author's results have, however, been called into question by recent work of Sigwart Ludke and Polano. According to these observers hæmolytic power does not properly belong to any one variety of streptococcus, but, on the contrary, frequent changes occur from the hæmolytic to the non-hæmolytic types, and *vice versâ*. Moreover, although a long-chained hæmolytic variety is usually met with in infected parturient women, certain fatal cases are due to micro-organisms with entirely opposite characteristics. Again, a streptococcus of a seemingly virulent type is met with in the lochia of healthy women at times. It is therefore impossible to draw any inferences from Schottlander's method, either as to the identification of varieties of streptococci or as to their virulence. The only element of bacteriological prognosis in puerperal infection which we possess at the moment would seem therefore to be the presence in the blood of streptococci, be they hæmolytic or not.

### Vaginal Thrush.

Vasilescu in the Spitalui reports several cases of Vaginal Infection due to the presence of Oïdium Albicans. The white membrane-like deposit has a great resemblance to that of diphtheria, but cultural methods allow of a correct diagnosis being made. Experiments with animals show that this fungus is by no means innocuous and that it may develop pathogenic properties. Intravenous injections of bouillon cultures caused death in from twenty to thirty hours in the case of rabbits, and the oïdium was found, after death, in pure culture in all the organs, including the bladder.

### Medicinal Treatment of Diabetes.

The latest suggestion for the Medicinal Treatment of Diabetes is one proposed by Dr. H. E. Smith in the *Practitioner*. In 1907 he had under treatment a very intractable case of this malady, complicated by ulcers of the leg and vulva. After trying many of the usual drugs he ordered iodide of calcium in five-grain doses thrice daily, and coincidently the patient's ulcers began to heal and the sugar was gradually reduced in three months from 8 per cent. to ½ per cent. At the same time the patient felt much better in her general health. Later on she had a relapse, which yielded under the same drug; ultimately the percentage of sugar fell after five months' administration to ¼ per cent., and the patient is still in good health with no symptoms which trouble her. Encouraged by this result the author has treated sixteen more cases in the same way, with almost exactly similar results as far as the percentage of sugar is concerned. The largest dose which has been given is fifteen grains three times a day, and many of the cases had previously been treated with codeine and other anti-diabetic remedies without benefit. No claim is made that the drug will "cure" all cases of diabetes; but certainly a powerful claim for further experiment has been substantiated. It is particularly noteworthy that the wretchedness, depression, and feelings of "illness" which many diabetics suffer from are greatly relieved by the treatment.

### Amaas.

An interesting paper on a Local Epidemic Disease, which is known in the district of Beaufort West, Cape Colony, as Amaas, is contributed to the *South African Medical Record* by Dr. J. P. Grant, who has seen 165 cases in four epidemics. The name is, of course, a native one, and the disease has been confused with chicken-pox, scabies, syphilis, pemphigus, measles, and acne. At one time the author believed that it had nothing to do with smallpox, but as the result of his extensive experience he is now convinced that it is a modified form of this disease. The cases are not so severe as ordinary smallpox, but several have been seen which have been more severe than varioloid—smallpox modified by previous vaccination. When this contention has been put forward in the past, various objections have been usually raised, but these Dr. Grant is now prepared to meet. Thus it has been said that Europeans are immune to amaas: but he has seen eight cases in Europeans, but two of whom had been vaccinated, and even then only in infancy. Then scarring is not very common in amaas: neither is it, urges the author, in mild and modified smallpox. The death-rate from amaas is about 5 per cent., which has been held to tend against its identity with smallpox: but then it is evidently an unusually mild variety of variola, so this low rate is not astonishing. On the other hand, the mode of onset of the disease, the distribution and nature of the rash, the pyrexia, and the comparative immunity of those who have been vaccinated (*i.e.* chiefly the white population) are all absolutely in accordance with the smallpox theory which Dr. Grant supports.

### X-Rays and Deeper Tissues.

At a recent meeting of the Académie des Sciences Drs. Regaud and Nogier made an interesting report on their experiments with *x*-rays to test their effects upon the deeper tissues. They find that the hard rays deprived of the soft rays (less penetrating) by filtration through a plate of platinum exercise a more energetic and selective action on the spermatogenic cells of the rat than the mixed rays. They state that they have found it possible to sterilise absolutely and completely the testicles of rats by means of a single application of the filtrated rays without producing any cutaneous changes. On the assumption that a malignant neoplasm would be as vulnerable to the rays as the spermatogenic cells of the rat, this same effect would be produced upon a neoplasm at a depth of 1.5 to 2 centimetres.

### Myasthenia following Influenza.

Drs. G. Ghedini and A. Fedeli have carried out some interesting researches on the myasthenic condition following influenza. They find that the curve of voluntary muscular work in patients recovering from influenza is shorter and lower than that of a normal individual or than that of a patient suffering from some acute affection other than influenza. They find, further, that in cases of influenza the myasthenic reaction hardly ever fails. It is generally well marked and the muscular tone is usually considerably diminished. All these phenomena steadily tend to disappear during convalescence. They have also carried out a series of experiments on rabbits. The toxins and antitoxins of the influenza bacillus were injected into the veins, the peritoneum and the muscles, and the condition of the gastrocnemius muscles studied by means of the myograph and ergograph. By these means they find that the myasthenic phenomena are due directly to the action of the influenza toxins and endotoxins, acting chiefly upon the nervous system.

### Projecting Ears.

Dr. E. Ruttin describes his method of operation for the Correction of Projecting Ears in the *Wiener Klinische Wochenschrift.* According to this author, excision of the cartilage may be necessary in some cases, but can usually be avoided. It has the disadvantage that injury of the cartilage may be followed by perichondritis and resulting deformity. The method advised by Dr. Ruttin consists in first determining, by placing the auricle against the side of the head, how much skin would be required to cover the posterior portion of the auricle after the ear has been fixed in a correct position. This is outlined by appropriate markings, and a bow-shaped incision is then made about 1 cm. behind the marked line, taking in the length of the auricle. Parallel with the first incision another curved cut is made, and the portion of skin between the two incisions is excised. The skin on the back of the auricle for a width of about 1 cm., if necessary as far as the helix, is then mobilised. The margins of the two cuts are approximated and sutured. In order to secure accurate apposition it may be necessary to supplement this procedure by excision of wedge-shaped portions of skin. A compress and bandage are applied and allowed to remain for fourteen days until superficial adhesion has taken place.

### Purulent Peritonitis.

The treatment of the Peritoneum after Opening the Abdomen in Cases of Purulent Peritonitis is a vexed question among surgeons. Some advocate a thorough cleansing and irrigation, while others prefer to cause as little disturbance and shock as possible, and depend a good deal upon the natural powers of resistance of the tissues to the further invasion of the microbes. The divergence of opinion arises a good deal from the difficulty of cleansing the peritoneal cavity owing to the complicated folds and twists of its contents. It is generally recognised, however, that every fibrinous or hæmorrhagic coagulum forms a field of culture for the bacteria which may be present. The simple manœuvre adopted by Dr. Kochs to get rid of such coagula appears worthy of mention. Having evacuated the septic contents of the abdomen he turns the patient over first on one side and then on the other, as far as possible, so as almost to make the patient lie on his abdomen. At the same time he raises successively first the chest and then the pelvis. By this means he has always succeeded in making many fibrinous flakes and clots escape, which would otherwise have remained in the abdomen, and he is assured that in this way he has often obtained a successful issue to the operation, which would otherwise have failed to relieve the condition.

### Urgent Dyspnœa in Children.

In a recent number of the *Guy's Hospital Gazette* Dr. H. C. Mann reports two interesting cases of urgent dyspnœa in children due to tuberculous mediastinitis from which spinal caries developed. The first case was under his care at the Evelina Hospital, and was a boy of eight, who was admitted for attacks of breathlessness. On percussion a dull note was obtained in the first and second intercostal spaces near the sternum on the right and to a less degree on the left. The neck was swollen, with enlarged veins visible in front over the clavicles and lower part of the neck. The episternal notch was obliterated, and the thyroid gland somewhat enlarged. Nothing abnormal was to be discovered on laryngeal examination. It was noted that the stridor was markedly increased when the head was flexed towards the sternum, and the boy complained of pain in the chest when in this position. He had no pain in the back, and the cervical spine appeared normal. One or two glands below the angle of the mandible were somewhat enlarged on the right side; there was no sign of a retropharyngeal abscess. Owing to the rapid increase in the severity of the symptoms, a diagnosis of mediastinal growth was made, probably originating in the thymus. The dyspnœa was very severe, and on two occasions the boy actually stopped breathing, and it was decided to perform tracheotomy, and push a catheter down through the bronchus. However, he recovered from the attacks, and the paroxysms of dyspnœa became less urgent. A fortnight later he complained of pain in the back, and tenderness was found over the last cervical and upper two dorsal vertebræ; and finally well-marked kyphosis developed. From the time spinal trouble became apparent his pain diminished, and the attacks of dyspnœa were less frequent, and eventually disappeared.

The second case was a boy of three years who had well-marked spinal caries with complete paraplegia, and developed urgent dyspnœa which came on suddenly and was accompanied with sucking in of the lower three intercostal spaces. On percussion there was a dull note over the upper part of the sternum and an impaired note over the upper two intercostal spaces near the sternum. During one of these attacks the child died. At the autopsy a large mediastinal abscess was found surrounding the œsophagus, and pushing down the trachea. This was densely matted to the surrounding structures, the remains of the thymus being involved in an abscess of the anterior mediastinum. The mass of inflammatory tissue flattened the trachea antero-posteriorly, and obstructed the innominate veins and the superior vena-cava. The bronchi were matted to the adjoining pulmonary vessels and cellular tissue, the mucosa of the lower part of the trachea and bronchi was œdematous and congested, the larger tubes containing sanguineous but not purulent secretion. There was no evidence of rupture into either bronchi or trachea, these structures being suronded by a multilocular abscess cavity evidently formed of breaking-down mediastinal glands, and not in communication with the abscess in the posterior mediastinum, which opens into the spinal canal through the remains of the body of the third dorsal vertebra. The second, third, and fourth dorsal vertebræ were all extensively involved with tuberculous caries, and the spine partially dislocated.

### Epigastric Pain.

Guinard and Lapeyre recently reported to the Paris Surgical Society the case of a man of 64 admitted to hospital for Epigastric Pain, which was characterised by violent attacks radiating to the shoulders. The patient had lost flesh lately and his skin was of a swarthy colour. Examination revealed a smooth, rounded tumour, very mobile, especially in a transverse direction, and dull to percussion. This dulness merged into that of the spleen. There was no ascites, nor could a movable kidney be made out. The urine showed no abnormality. On opening the abdomen a dark-coloured mass was found which burst in process of manipulation, setting free about three pints of blood-clot. This tumour lay in the cavity of the pancreas behind the lesser omentum, and reached to the tail of the pancreas. The kidney and spleen were intact. The cavity in which lay the tumour was plugged and the patient sent back to bed. In spite of all attention he, however, died the same night. The authors regret that a preliminary distension of the stomach with gas was not performed, as this would undoubtedly have allowed of a correct diagnosis of post-stomachal or pre-pancreatic tumour being made.

### Primary Erysipelas of the Larynx.

An interesting case of Primary Erysipelas of the Larynx is reported by G. Solaro in Il Morgagni. A workman, aged 46, was seized with severe chills and prostration, accompanied by hoarseness, burning pains in the throat, and dyspnœa. On admission into hospital his temperature was 104° F. and his pulse rate 110. The submaxillary and cervical glands were enlarged. The mucous membrane of the larynx was red and swollen, this condition being especially well marked in the region of the false vocal chords, so that the laryngeal meatus was much reduced in size. It was found necessary to relieve the dyspnœa by tracheotomy, which was performed under stovaine, a tube being inserted. Subsequently the erysipelatous condition spread to the skin of the whole of the anterior surface of the neck, but in a few days the fever disappeared and the cutaneous and laryngeal conditions rapidly improved. It was not possible, however, to remove the tube until two weeks after the operation, owing to the persistence of the swelling of the false vocal chords. The patient eventually made a good recovery, and some months later a laryngeal examination failed to reveal anything abnormal.

